# A comparison between an ICT tool and a traditional physical measure for frailty evaluation in older adults

**DOI:** 10.1186/s12877-019-1089-z

**Published:** 2019-03-21

**Authors:** Anna Mulasso, Paolo Riccardo Brustio, Alberto Rainoldi, Gianluca Zia, Luca Feletti, Aurèle N’dja, Susanna Del Signore, Eleonora Poggiogalle, Federica Luisi, Lorenzo Maria Donini

**Affiliations:** 1 NeuroMuscular Function Research Group, School of Exercise and Sport Sciences, Department of Medical Sciences, University of Torino, Torino, Italy; 2 Caretek s.r.l, Turin, Italy; 3 Sanofi-Aventis R&D, Chilly-Mazarin, France; 4 Bluecompanion ltd, London, UK; 5 Food Science and Human Nutrition Research Unit, Department of Experimental Medicine, Sapienza University, Rome, Italy

**Keywords:** ICT tool, Physical measure, Physical functioning, Health status, Sarcopenia

## Abstract

**Background:**

Frailty is a clinical condition among older adults defined as the loss of resources in one or more domains (i.e., physical, psychological and social domains) of individual functioning. In frail subjects emergency situations and mobility levels need to be carefully monitored. This study aimed to: i) evaluate differences in the mobility index (MI) provided by ADAMO system, an innovative remote monitoring device for older adults; ii) compare the association of the MI and a traditional physical measure with frailty.

**Methods:**

Twenty-five community-dwelling older adults (71 ± 6 years; 60% women) wore ADAMO continuously for a week. The time percentage spent in Low, Moderate and Vigorous Activities was assessed using ADAMO system. Walking ability and frailty were measured using the 400 m walk test and the Tilburg Frailty Indicator, respectively.

**Results:**

Controlling for age and gender, the ANCOVA showed that frail and robust participants were different for Low (frail = 58.8%, robust = 42.0%, *p* < 0.001), Moderate (frail = 25.5%, robust = 33.8%, *p* = 0.008), and Vigorous Activity (frail = 15.7%, robust = 24.2%, *p* = 0.035). Using cluster analysis, participants were divided into two groups, one with higher and one with lower mobility. Controlling for age and gender, linear regression showed that the MI clusters were associated with total (β = 0.571, *p* = 0.002), physical (β = 0.381, *p* = 0.031) and social (β = 0.652, p < 0.001) frailty; and the 400 m walk test was just associated with total (β = 0.404, *p* = 0.043) and physical frailty (β = 0.668, p = 0.002).

**Conclusion:**

ADAMO system seems to be a suitable time tracking that allows to measure mobility levels in a non-intrusive way providing wider information on individual health status and specifically on frailty. For the frail individuals with an important loss of resources in physical domain, this innovative device may represent a considerable help in preventing physical consequences and in monitoring functional status.

## Background

Population ageing is transforming the demographic structure in Europe [1] and in the World [2] and this change is recognized as one of the most significant public challenges in term of health care and social problems [3]. Thus, preventive and health promotion strategies have been pursued in order to encourage both a healthy ageing and an independent living.

Information and communication technologies (ICTs) may be an innovative and pervasive tool to monitor and evaluate health domains in older adults during everyday life activity [4, 5]. For example, telemonitoring (i.e., the use of audio, video and other telecommunication technologies to monitor health status at distance) may reduce the possible negative outcomes, such as emergency visits or hospitalisations [6]. The use of ICTs seems to improve also the quality of life, the social interaction and the general wellbeing of older adults [7, 8].

ADAMO system (Caretek S.r.l., Turin, Italy) is an innovative remote monitoring device for older adults. Briefly, ADAMO system is composed of a Base Station, installed at user’s home, receiving data from a carewatch worn by the same user. Wearing the carewatch during every daily life activities, ADAMO system is able to monitor user’s indoor and outdoor activity, by signalling any suspicious immobility, any falls followed by lack of movement and mobility index (MI) levels. Specifically, the carewatch records raw data from its sensors (i.e., triaxial accelerometer sampling at 50 Hz) and, every 10 min, transmits them to the Base Station, which forwards such an information to the storage server. ADAMO system was specifically designed for older adults and demonstrated to measure the number of steps in walking activity accurately, in particular for slow walking speed [9], typical of older adults and specific populations, such as frail older adults [10].

Frailty is a clinical condition among older adults defined as the loss of resource in one or more domains (i.e., physical, psychological and social domains) of individual functioning [11]. Senile anorexia and malnutrition have to be considered among the main risk factors for frailty. Authors [12, 13] underline that the risk of frailty may be reduced by improved nutritional status for macronutrients and micronutrients. Frailty makes older adults more vulnerable and susceptible to adverse health outcomes, such as falls, hospitalization, institutionalization and mortality [14]. An impaired physical function is the major indicator of frailty [14] and negatively affects the autonomy in the activities of daily living. In particular, the mobility function, defined as the ability to move in the environment easily and without restriction [15], resulted strictly related to frailty [14]. Indeed, poor mobility was associated with higher frailty scores [16]. Moreover, the daily step count and the amount of energy spent performing the activities of daily living (in kcal/week) were strongly associated with frailty levels [17].

Generally, mobility function, based on self-report or performance-based measurements, are useful tools for frailty screening (e.g., [16, 18–21]). However, these measures may lead to possible self-report bias, non-objective parameters [22] or may be not ecological. For example, a Walking test, that evaluates the time taken to walk a path, typically is used to assess mobility function in older adults (e.g., [23, 24]). However, this assessment is limited to laboratory environment and does not reflect the mobility in everyday life activity. Thus, ICT systems may provide objective parameters for monitoring continuously and in a non-intrusive way the older adults during everyday life. Indeed, ICT systems that evaluates mobility levels (e.g., resting, sitting or walking activity) during everyday life activity may overcome the limitations of the traditional assessment of older adults’ health status and provide specific information on their behaviour [25, 26].

## Methods

### Study aims

To the best of our knowledge no study compared physical screening tools and an ICT system aimed to measure the mobility level for evaluating frailty in older adults. Therefore, the purpose of this study was to investigate the relationships between the mobility index (MI) provided by ADAMO system and the frailty level in a group of Italian community-dwelling older adults. Specifically, the aims of the study were: (1) to test differences in the MI levels of frail and robust individuals, and (2) to compare the association of the MI and a traditional physical measurement – the 400 m walk test - with frailty (total, physical, psychological and social frailty). We hypothesized that ADAMO system may be useful to evaluate differences in the MI levels for frail and robust individuals. Moreover, as ADAMO system is worn during everyday life activity and not in a laboratory setting, we think that it may provide additional and more exhaustive information about older adults’ general health status.

### Design and study population

The development and validation of ADAMO system is part of the SPRINTT project (Sarcopenia & physical frailty in older people: multi-component treatment strategies; 9th Call IMI 2013) aimed to validate and implement a practical and clinical prevention of physical frailty and sarcopenia and its complications [27, 28] (http://www.mysprintt.eu/en) [29]. However, this current paper does not report direct data collected from SPRINTT project rather is based on data recorded during an IMI-SPRINTT ancillary Living Lab designed to test the acceptance of the ADAMO carewatch for physical activity measurement.

### Participants

Among the 35 persons contacted, 25 (71.4%) were enrolled in this study. Specifically, 5 (14.3%) did not meet the study inclusion criteria and 5 (14.3%) did not agree to participate. Recruitment of the study participants was conducted in April 2017 in two sites (Turin and Rome) and the data collection was performed in the period of May/June 2017.

Participants satisfying all of the following criteria were included: i) they were aged over 65 years; ii) they agreed to test a connected device and to wear it continuously on 7-day duration; iii) they were able to come to the test centre by themselves or accompanied by a family member; iv) they were able to understand and answer the study questionnaires; v) they could walk independently with or without the use of assistive devices. Individuals with any acute diseases (i.e., recent fractures or surgical operation) and/or chronic diseases (i.e., dialysis, respiratory insufficiency, coronary disease, known myocardiopathies, severe osteoarthritis) preventing the practical requirements for study participation or the administration of physical test were excluded.

The study protocol was approved in the context of the IMI (Innovative Medicine Initiative) in the grant agreement No 115621 Sarcopenia and Physical fRailty IN older people: multi-componenT Treatment strategies – SPRINTT – and Amendment No 2, 22 May 2015. The study was performed in accordance with the Declaration of Helsinki. All the participants signed their written informed consent statement in conformity with Italian law and the ethical code of the American Psychological Association. No rewards or incentives for participating were provided.

### Procedure

At day one, participants came to the test centre to complete preliminary procedures. Firstly, they have been informed about the device and the protocol, they signed the informed consent and they were screened in accordance with inclusion criteria; secondly, they received the questionnaire to be autonomously filled out at home and collected ADAMO carewatch with detailed instructions about its activation; thirdly, they performed the physical test (individually for each participant and in the presence of an expert in exercise and sport sciences). Completing the questionnaire took on an average of 10 min. Approximately, the same time-span was required to perform the physical test. During the week, all the participants received two phone calls: on day two to verify if there were any problems or doubts related to the use of device, and on day six to remember them to fill out the questionnaire and to make an appointment to pick the device up. The appointment was fixed starting from day eight directly at participants’ home. On the occasion of the appointment, the operator asked about the encountered doubts in completing the questionnaire and he/she checked for any missing answer.

### Measures

The mobility index (MI) referred throughout the 7-days was obtained from ADAMO web service. The MI is a parameter that explains the amount of physical activity performed by the user, providing information about the amount of time spent lying or sitting, standing still or walking with different intensity. The MI is computed by processing the acceleration pattern on the three axes and taking into account the detected number of steps. The MI provides the percentage of time spent throughout 7-days in each of the following levels: (i) Very Low Mobility: user is lying or sitting while resting (e.g., sleeping, sitting); (ii) Low Mobility: user is lying or sitting performing a slight activity (e.g., sitting having a meal, playing cards, performing leisure activities in front of television); (iii) Medium Mobility: user is still standing or walking with a reduced intensity (e.g., cooking, ironing); (iv) High Mobility: user is walking with a normal intensity; and (v) Very High Mobility: user is walking with a sustained pace. For each individual, the total amount of these levels returns a score of 100%.

Walking ability was measured by the 400 m walk test [30]. Participants were instructed to walk 8 laps (50 m per lap) along a corridor inside the test centre building at their usual pace without overexerting themselves. The total time (s) for completing the test was collected using a manual chronometer. If a participant referred of having chest pain or dyspnea, the test was immediately stopped. The use of assistive devices was allowed during the walk.

Frailty was evaluated using the part B of the Italian version of the questionnaire Tilburg Frailty Indicator (TFI: [31, 32]). The part B of the TFI is composed of 15 items related to three domains (physical, psychological and social) of human functioning [31]. The physical domain comprises eight items about physical activity, unexplained weight loss, walking problems, difficulty in balance, limited vision, hearing problems, strength in hands and physical tiredness. The psychological domain consists of four questions on cognition, depression, anxiety and coping. The last three items belong to the social domain and are related to living alone, social relations and social support. The part B ranged from 0 (absence of frailty) to 15 (severe frailty), with a cut-off value equal or higher than 5 that classifies frail from robust individuals [32].

Socio-demographic characteristics (i.e., age, gender, marital status and level of education) and health condition status (i.e., usual use some drugs and the presence of one or more chronic diseases) were self-reported.

### Statistical analysis

Descriptive analyses were carried out for all the study variables. Based on the data provided by ADAMO, the following three continuous variables were obtained and used for statistical analyses:

- Low Activity: percentage of time spent in Very Low Mobility activity;
- Moderate Activity: sum of time percentages spent in Low and Moderate Mobility activity;
- Vigorous Activity: sum of time percentages spent in High and Very High Mobility activity.

In other words, the first two levels consisted of lying, sitting or performing light activities activities, while the other level consisted of walking activities at usual or sustained pace. For each individual, the sum of Low, Moderate and Vigorous Activities variables returns a score of 100%.

Firstly, controlling for age and gender, one-way analysis of covariance (ANCOVA) was performed to investigate differences in Low, Moderate, and Vigorous Activities between frail and robust individuals. Secondly, cluster analysis was carried out to detect participants with similar levels of Low, Moderate and Vigorous Activities. Specifically, the following two steps was performed: (i) hierarchical cluster procedure using Ward’s method to individuate the appropriate number of clusters by observing the dendrogram; (ii) k-means clustering to partition individuals into k homogenous groups. Using one-way analysis of variance significant differences across the cluster centers were identified. Lastly, to test the association of clusters based on the MI levels and the 400 m walk test with frailty – total, physical, psychological, and social frailty – linear regression analysis, controlling for participants’ age and gender, was carried out. The Statistical Package for Social Sciences, version 24.0 (SPSS Inc., Chicago, IL, USA) was used for the analyses. The level of significance was set at *p* < 0.05.

## Results

### Participants characteristics

Table 1 shows the characteristics of the study participants. The mean age was 71 years (SD =6 years; range 65–89 years) and most were women (60%), married (52%), and with a level of education corresponding to secondary school (32%) or to high school diploma (36%). A high number of participants referred to have one or more chronic diseases (60%) and to consume drugs regularly (68%).

**Table 1:** Characteristics of participants (*N* = 25)

Variables	n (%)	M (SD)
Age, years		71 (6)
Gender, number of women	15 (60)	
BMI, kg/m^2^		29.4 (7.6)
Marital status		
Married	13 (52)	
Not married	3 (12)	
Widowed	9 (36)	
Level of education		
Primary school, 5 years	5 (20)	
Secondary school, 8 years	8 (32)	
High school diploma, 13 years	9 (36)	
University degree, 18 years	3 (12)	
Chronic disease, number of Yes	15 (60)	
Pharmacotherapy, number of Yes	17 (68)	
MI provided by ADAMO, %		
Low Activity		51.4 (11.2)
Moderate Activity		29.2 (9.8)
Vigorous Activity		19.4 (9.7)
400 m walk test, s		326 (93)
TFI, points		5 (5)
Physical TFI		2 (1)
Psychological TFI		1 (1)
Social TFI		1 (1)
Level of frailty ^a^		
Frail persons	14 (56)	
Robust persons	11 (44)	

*Note*s: ^a^ Individuals with a score equal or higher than 5 were classified frail. M, mean; SD, Standard Deviation; BMI, Body Mass Index; TFI, Tilburg Frailty Indicator, higher score corresponded to more severe frailty condition; MI, Mobility index

The means percentage of time spent in Low, Moderate and Vigorous Activities were 51.4% (SD = 11.2%), 29.2% (SD = 9.8%), and 19.4% (SD = 9.7%), respectively. On average, participants completed the 400 m walk test in 326 s (SD = 93 s; range 204–614 s). The mean TFI total score was 4.8 points (SD = 2.3 points; range 0–9 points). Overall, 14 (56%) of 25 individuals were categorized as frail (TFI score ≥ 5 points).

### Differences in MI levels between robust and frail individuals

Significant differences between robust and frail individuals were observed for Low Activity [F (1,21) = 40.3, *p* < 0.001], Moderate Activity [F(1,21) = 8.6, *p* = 0.008], and Vigorous Activity [F (1,21) = 5.1, *p* = 0.035]. See Table 2.

**Table 2:** Differences of ADAMO mobility levels for frailty status

	Group		F (1,21)	Effect Size	p
	Robust	Frail			
	M (SD)	M (SD)			
Continuous Variables					
Low Activity	58.8 (6.6)	42.0 (8.3)	40.3	0.657	<0.001
Moderate Activity	25.5 (7.6)	33.8 (10.6)	8.6	0.292	0.008
Vigorous Activity	15.7 (7.2)	24.2 (10.8)	5.1	0.195	0.035

*Notes: M* mean, *SD* Standard Deviation

### Relationship of the MI levels and the 400 m walk test with frailty

The hierarchical cluster analysis highlighted two clusters. The cluster centers for Low Activity (p < 0.001) and Moderate Activity (*p* = 0.001) were statistically different from each other. On the contrary, the cluster centers for Vigorous Activity did not show significant differences between the two clusters. The first cluster (called “Good MI”) consists of 12 individuals, characterized by low percentage of Low Activity associated with high level of Moderate and Vigorous Activity. The second cluster (called “Low MI”) includes 13 individuals, with a high level of Low Activity and low level of Moderate and Vigorous Activity. See Table 3.

**Table 3:** Clusters based on ADAMO mobility index

ADAMO mobility index	Clusters		p^a^
	1 – Good MI	2 – Low MI	
Low Activity	42.2	59.9	<0.001
Moderate Activity	35.4	23.5	0.001
Vigorous Activity	22.4	16.6	0.142
Number of cases	12	13	–

*Notes:* ^a^ Results based on one-way ANOVA; MI, Mobility Index

Linear regression analysis, controlling for age and gender, showed that both clusters based on the MI and the 400 m walk test were associated with total and physical frailty. In particular, both clusters based on MI and 400 m walk test significantly predicted total (β = 0.571, *p* = 0.002 and β = 0.404, *p* = 0.043) and physical frailty (β = 0.381, *p* = 0.031 and β = 0.668, p = 0.002). Clusters based on the MI were more strongly associated with total frailty, while the 400 m walk test with physical frailty. These two models explained 35.2 and 35.7% of variance, respectively. Differently, only the clusters based on the MI were associated with social frailty (β = 0.662, *p* < 0.001), explaining 48.3% of variance. Finally, no statistical significant associations with psychological frailty resulted. For more details see Table 4.

**Table 4:** Relationship of ADAMO mobility index and the 400 m walk test with frailty

Components of physical frailty	TFI				Physical TFI				Psychological TFI				Social TFI			
	B	SE	β	p	B	SE	β	p	B	SE	β	p	B	SE	β	p
Clusters based on MI	2.627	0.760	0.571	0.002	1.078	0.466	0.381	0.031	0.447	0.330	0.277	0.191	1.102	0.249	0.652	< 0.001
400 m walk test	0.010	0.005	0.404	0.043	0.010	0.003	0.668	0.002	0.001	0.002	0.151	0.523	−0.002	0.002	−0.163	0.342

*Notes:* Analyses were adjusted for age and gender. TFI, Tilburg Frailty Indicator, higher score corresponded to severe frailty condition; SE, Standard Error; MI, Mobility Index

## Discussion

The present study was designed to investigate the relationships between the MI levels provided by ADAMO system and a mobility screening tool with frailty in a sample of Italian community-dwelling older adults. To this end, we compared the MI levels for frail and robust individuals and investigated the association of the clusters based on the MI levels and the 400 m walk test with frailty (i.e., total, physical, psychological and social domains).

Our results showed that frail individuals reported different MI levels compared with robust older adults depending on intensity of daily activities. Indeed, frail individuals showed higher percentage of time spent in Low Activity (i.e., lying or sitting activities while resting) and lower percentage of time spent in Low Moderate Activity (i.e., slight activity while lying or sitting and standing or walking activity with reduced intensity) and in Vigorous Activity (i.e., walking with normal intensity or with a certain intensity). Interesting, data suggested a moderate effect size in the difference between frail and robust participants for Low Activity. In contrast, a small effect was observed in the differences between these groups for moderate and vigorous activity. Taken together, our results suggest that the differences between frail and robust participants are more evident in Low Activity levels rather than in Moderate or Vigorous Activities. Thus, we may speculate that frail older adults reduce the intensity of the execution of the activities of daily living and they spend more time in a condition of rest in comparison with robust older adults. Similar to our results, a study of Portegijs and colleagues [33] showed that mobility performances in different life-space levels (e.g., bedroom, other rooms, outside home, neighborhood, town, beyond) were negatively affected by frailty status. Another study of Schwenk and colleagues [22] demonstrated that parameters related to mobility discriminated frailty. Specifically, the daily percentage of mobility and sitting time, the number of maximum continuous steps, the walk bouts mean duration and the longest walking bout duration were all statistically different between frail and robust older adults with better results for robust than frail people. Therefore it is possible to assume that mobility restriction in frail older adults might be due to the loss of reserve in one or more frailty domains. Physical frailty may be considered a geriatric syndrome characterized by progressive and generalized sarcopenia with an increased risk of physical disability, poor quality of life and death [34]. For example, an impairment in physical functioning (e.g., balance or gait dysfunctions), an increased exhaustion perception, and/or the onset of vision or hearing problems that are key indicators of physical frailty can strongly impact on individual mobility [35]. As well indicators of psychological and social frailty domains, such as depressive symptoms, anxiety perception and/or loss of social relationships can affect mobility in older adults [36, 37]. Special attention should be drawn to the prevention of each component of frailty with, for example, the implementation of multidomain interventions specific for the compromised domain.

Moreover we found that both the clusters based on the MI levels and the 400 m walk test were associated with total and physical frailty. Interestingly the clusters based on the MI levels were strongly associated with total frailty rather than the 400 m walk test. On the contrary, 400 m walk test was more strongly associated with the physical frailty compared to the clusters based on the MI levels. Differently, only clusters based on the MI levels were associated with social frailty. These are expected findings, since the MI levels reflect the mobility function in a continuous and non-intrusive way during everyday life. Consequently data on the MI levels are more informative and exhaustive than data provided by a traditional physical measure of functioning, such as the 400 m walk test, and probably they are able to capture the complex relationship and interrelationships among factors of different domains which can lead to frailty. On the contrary, the 400 m walk test is a measure limited to laboratory setting and it seems to be more indicated to provide information just on physical health status of individuals and during periodic assessment. These results seems to be interesting and promising since functional assessment is important to evaluate the physical consequences of sarcopenia and malnutrition that are often linked to frailty.

Some limitations should be underlined. First of all, the small sample size did not allow us to generalize the results. As a consequence, also the two clusters to discover mobility profiles among individuals included a limited number of participants. A larger sample size should be considered to improve the causal interpretation of our results. Additionally, based on the Mobility index provided by ADAMO system we arbitrarily created three continuous variables: Low Activity (percentage of time spent in very low mobility activity), Moderate Activity (sum of time percentages spent in low and medium mobility activity) and High Activity (sum of time percentages spent in high and very high mobility activity). Thus, caution is needed in interpreting these results. Furthermore, the cross-sectional nature of the study did not allow us to investigate the causal relationship between the MI levels and frailty domains over time. Due to the limitations of this study, future longitudinal studies are needed to deepen the causal mechanism linking the MI levels provided by ADAMO and frailty.

## Conclusions

In conclusion, results from this study indicate that ADAMO allows to measure mobility levels in a non-invasive way providing wider information related to individual general health condition, and specifically to frailty, in a population of older adults. It is worth noting that ADAMO is a time-saving tool and less stressful than traditional physical measures of functioning detected in a laboratory environment. Consequently, ADAMO may be an useful telemonitoring tool for older adults.

## Abbreviations

IMI: Innovative Medicines Initiative
ITC: Information and Communication Technology
MI: Mobility Index
SPRINTT: Sarcopenia and Physical fRailty IN older people: multi-componenT Treatment strategies
TFI: Tilburg Frailty Indicator
